# Case Report: Schwannoma of the sigmoid colon: a case report of a rare colonic neoplasm and review of literature

**DOI:** 10.12688/f1000research.19110.1

**Published:** 2019-05-13

**Authors:** Gangmi Kim, Sun Il Kim, Kang Young Lee

**Affiliations:** 1 Department of Surgery, Severance Hospital, Yonsei University College of Medicine, Seoul, 03722, South Korea; 2 Department of Pathology, Severance Hospital, Yonsei University College of Medicine, Seoul, 03722, South Korea

**Keywords:** Colon, schwannoma, colonic neoplasms, colectomy

## Abstract

**Background:** Schwannomas are tumors originating in Schwann cells of the peripheral nerve system and uncommonly develop in the gastrointestinal tract. Sigmoid colon schwannomas are very rare and only 28 cases have been reported. This study aims to report a case of a sigmoid colon schwannoma and present a literature review.

**Case report:** We report a case of a 66-year-old female with asymptomatic sigmoid colon schwannoma. The patient underwent a screening colonoscopy and about 4cm sized submucosal tumor was identified at the sigmoid colon. A colonoscopic biopsy was performed and the microscopic exam revealed an ulcerated lesion with a proliferation of fibroblast-like spindle cells beneath ulcer, which was insufficient for diagnosis. Abdominopelvic computerized tomography (CT) scan showed a well-defined, well-enhancing, round shaped and slightly heterogenous mass at the sigmoid colon. No distant metastasis was identified in abdominopelvic CT and chest CT scans. Carcinoembryonic antigen level was within a normal range (1.33ng/mL). The patient underwent laparoscopic anterior resection. Immunohistochemical staining of the resected specimen showed positivity for S-100 protein in tumor cells and schwannoma was diagnosed post-surgically. Surgical resection margins were free from tumor and no regional lymph node metastasis was reported.

**Conclusion:** Colon schwannomas are rare diseases. Most cases of colon schwannomas are accidentally identified during screening colonoscopy. The tumors usually present as submucosal masses and colonoscopic biopsies are mostly non-diagnostic. Surgical resection is required, and definitive diagnosis is made by confirming S-100 positive tumor cells in immunohistochemical analysis. Most cases are benign; a few cases have been reported to be malignant. Surgical resection with free negative margins is the treatment of choice

## Introduction

Schwannomas are a common type of tumor of peripheral nerve in adults which originate in Schwann cells. These tumors mainly present along the peripheral nerves and are rarely identified in the gastrointestinal (GI) tract ^1, 2^. GI tract schwannomas develop most frequently in the stomach (83%), and less frequently in the small intestine (12%), colon and rectum ^3^. Sigmoid colon schwannomas are very rare and only 28 cases have been reported so far ^3, 4^. Most colon schwannomas are incidentally identified as submucosal tumors on screening colonoscopy ^1, 5^. Colonoscopic biopsies alone usually provides limited information and definite diagnosis is made after surgical resection ^1^. Most of the colon schwannomas are benign and surgical resection with adequate free resection margins is the treatment of choice ^6^. Here we present a case of sigmoid colon schwannoma and discuss the clinical features of the disease with a literature review.

## Case report

**Figure 1.**
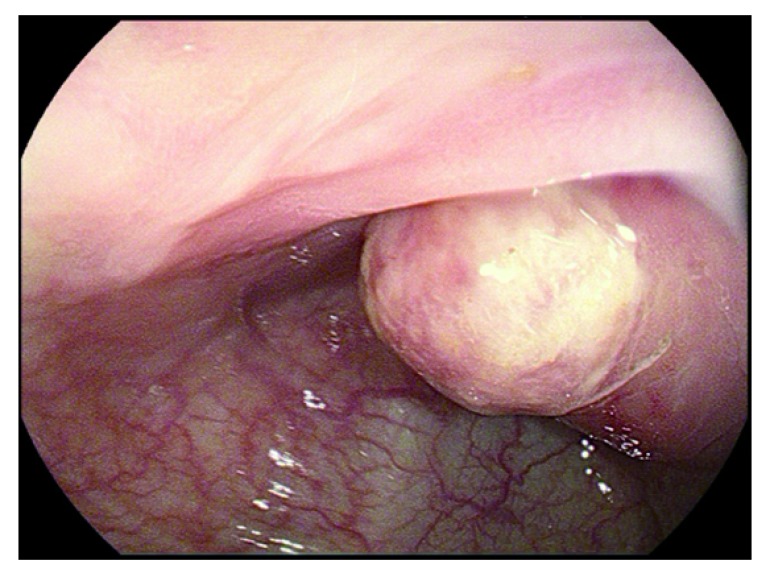
Colonoscopy. About 4cm sized submucosal tumor was identified at the sigmoid colon.

A 66-year-old Asian female patient, who was a housewife, visited a local clinic for a routine screening colonoscopy in mid-January 2018. During the colonoscopy, a submucosal tumor sized about 4cm was identified at the sigmoid colon ( Figure 1) and biopsy was performed. The microscopic exam of the biopsied specimen showed an ulcerated lesion with a proliferation of fibroblast-like spindle cells beneath the ulcer, which was insufficient for a definite diagnosis.

The patient was referred to our hospital at the end of January 2018. She presented no specific symptom and physical examination showed no specific finding. She had a history of hypertension and a benign breast mass. She had a positive family history of cancer: her father had gastric cancer, and her uncle had lung cancer.

**Figure 2.**
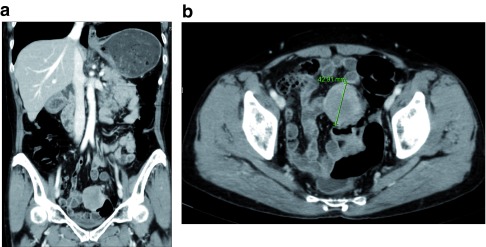
Abdominopelvic computerized tomography (CT). A well-circumscribed, well-enhanced, round-shaped mass was identified at the sigmoid colon. ( **A**) Coronal view. ( **B**) Axial view.

An abdominopelvic computerized tomography (CT) scan revealed 4.4cm sized well-circumscribed, well-enhanced, round-shaped mass in the sigmoid colon, which was slightly heterogenous inside. No intraabdominal metastasis was identified ( Figure 2A–2B). Chest CT scan showed no intrathoracic metastasis. Carcinoembryonic antigen (CEA) level was 1.33ng/mL, which was within a normal range (0.0–5.0ng/mL). Other laboratory test results were also within normal ranges. Differential diagnosis was 1) gastrointestinal stromal tumor (GIST); and 2) neuroendocrine tumor (NET).

The patient underwent a laparoscopic anterior resection. On laparoscopic exploration, an extruding mass was identified at the anterior wall of the sigmoid colon and no metastasis was observed. The sigmoid colon was mobilized and the inferior mesenteric artery was low ligated. Sigmoid colon resection with end-to-end anastomosis was performed.

**Figure 3.**
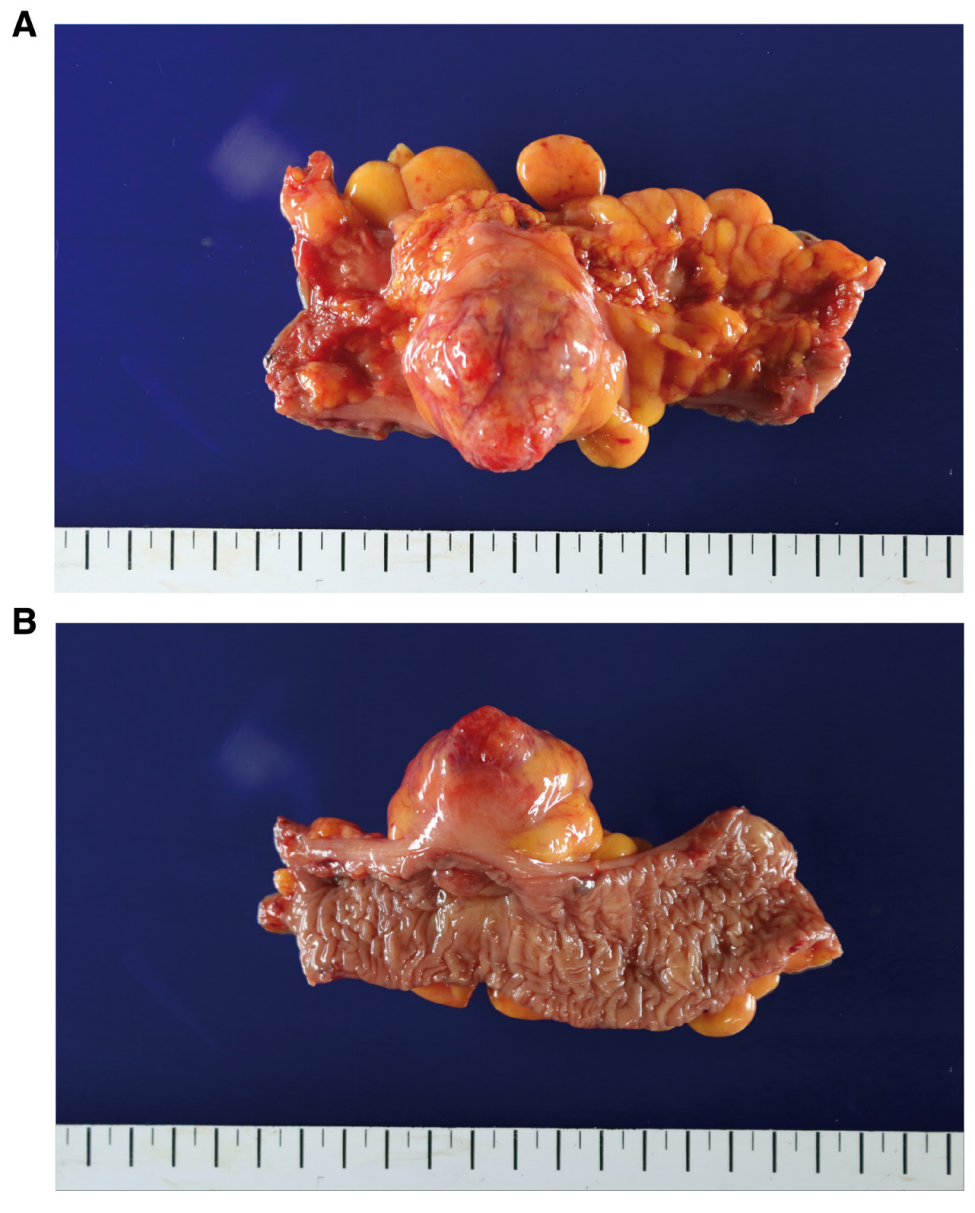
Gross findings of the resected specimen. ( **A**) 4.5 × 4.0cm sized round, protruding mass was observed on the surface of the serosa. ( **B**) The photo was taken from the mucosal side.

**Figure 4.**
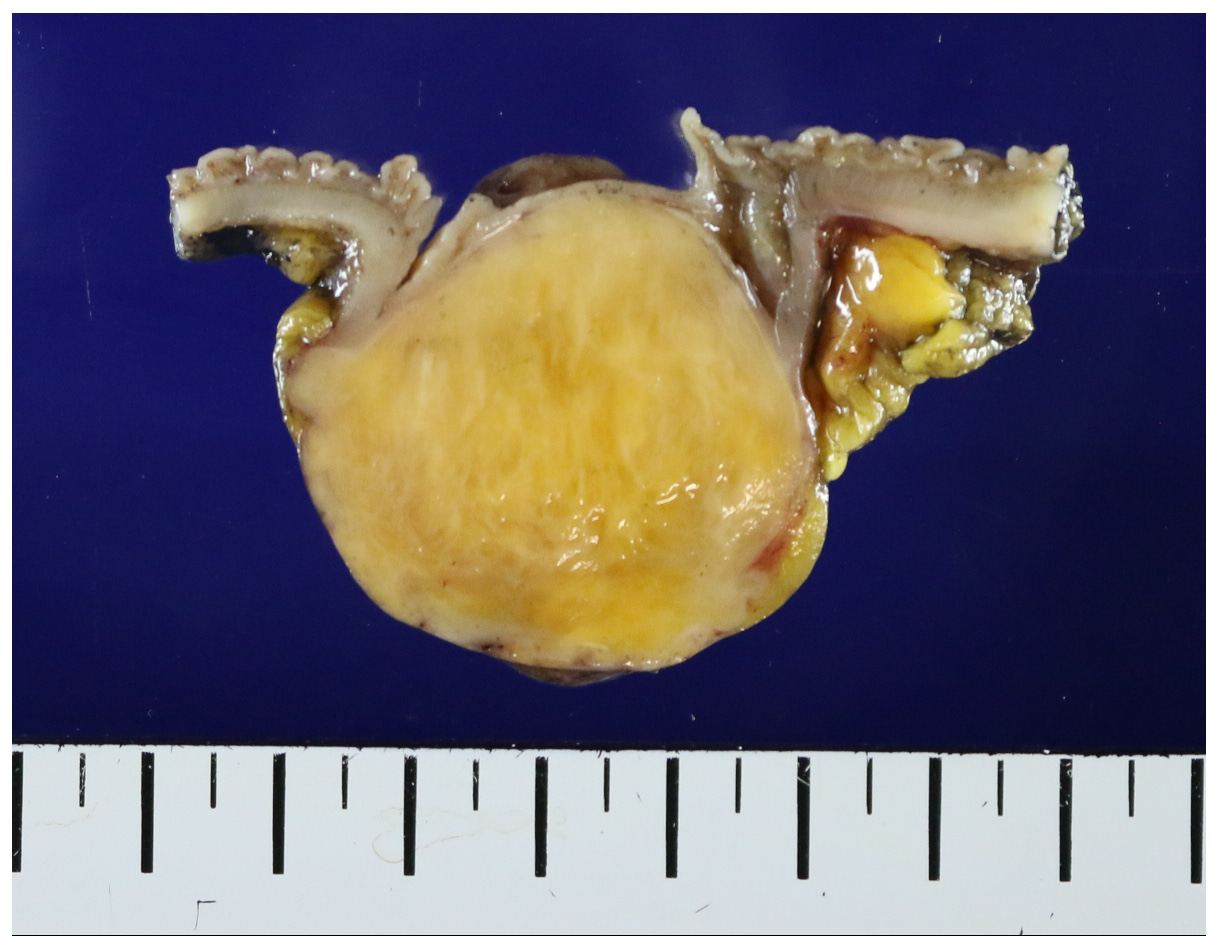
Cut section after fixation. Relatively well-demarcated yellowish mass without encapsulation is shown.

**Figure 5.**
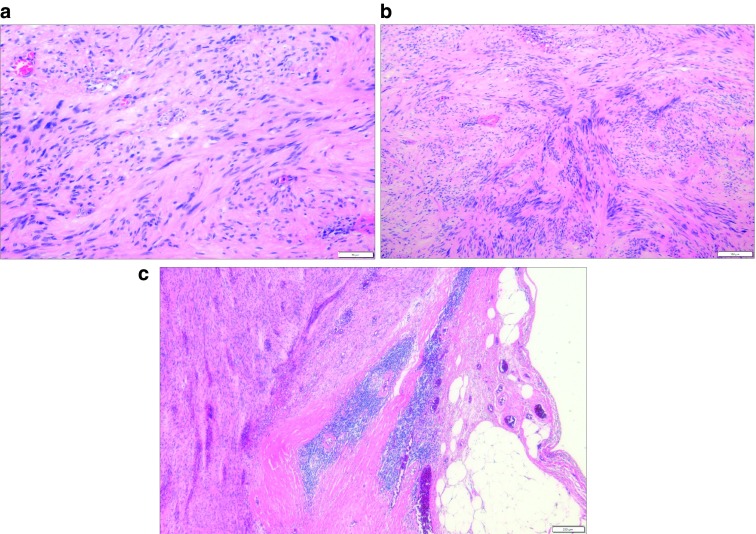
Hematoxylin and eosin (H&E) stain. ( **A**) The tumor cells are composed of spindle cells with low nuclear atypia. ( **B**) Nuclear palisading growth pattern is shown. ( **C**) Lymphoid cuffing surrounding tumor cells is shown.

**Figure 6.**
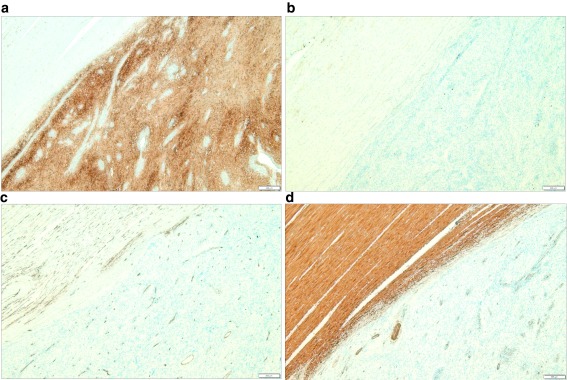
Immunohistochemical stain. ( **A**) S-100 is diffuse, strong positive in tumor cells. ( **B**) C-KIT is negative in tumor cells. ( **C**) CD34 is negative in tumor cell, but normal vessel structures were stained. ( **D**) SMA is negative in tumor cells, but normal smooth muscle in the proper muscle layer is stained.

On examining the resected specimen, about 4.5 × 4.0cm sized round mass was observed on the surface of the serosa and there was no tumor infiltration to the serosa ( Figure 3A–3B). The tumor was located 7cm from the proximal resection margin and 4cm from the distal resection margin. On sections after fixation, the cut surface showed a yellowish mass (4.2×3.2cm), which was abutting on the circumferential resection margin. The mass was relatively well-demarcated without encapsulation ( Figure 4). On hematoxylin and eosin (H&E) stain, the tumor was composed of spindle cells with low nuclear atypia, with nuclear palisading growth pattern, and lymphoid cuffing surrounding tumor cells were identified ( Figure 5A–5C). Mitosis was rarely observed (1/50 in high-power field). The remaining mucosa and serosa were grossly unremarkable. The resection margins were free from tumor. Lymph node metastasis was zero in 13 regional lymph nodes. On immunohistochemical analysis, s-100 was strongly positive in tumor cells; otherwise, c-kit, CD34, and SMA were negative ( Figure 6A–6D). Finally, the diagnosis was a benign schwannoma of the sigmoid colon.

The patient recovered from surgery uneventfully and was discharged on postoperative day 5. When she visited the out-patient clinic two weeks after discharge, she did not present any complication. No postoperative adjuvant therapy was performed.

## Discussion

Schwannomas are peripheral nerve sheath tumors which rarely develop in GI tract ^1, 2^. GI tract schwannomas represent about 2–6% of all mesenchymal tumors ^2, 3^.

For the first time, Daimaru *et al.* clarified the entity of the nerve sheath tumors developing in the GI tract and proposed these tumors to be designated as “benign schwannoma of the GI tract” in 1988 ^7^. Lymphoid cuffing, benign nuclear atypia and positive immunostaining for S-100 protein were the distinct features of the schwannoma of the GI tract, which distinguish the schwannoma from other spindle-cell stromal tumors of smooth muscle origin ^7^. Until the early 1990s, most GISTs traditionally had been classified as smooth muscle tumors ^8^. Ueyama *et al.* suggested that most of the GIST had smooth muscle differentiation and excluded schwannomas from the GIST ^8^.

Currently, GI tract schwannomas are classified as non-epithelial tumors of which disease entity is clearly distinct from leiomyomas, leiomyosarcomas, gastrointestinal autonomic nerve tumors (GANTs) and GISTs ^2, 9^. And GI schwannomas are considered distinguished from conventional soft-tissue schwannomas and CNS schwannomas ^2^.

GI schwannomas are mostly identified in the stomach and less frequently seen in colon, rectum, small intestine or esophagus ^2, 3^. They are most frequently diagnosed among people in their sixties and the incidence rates are identical for males and females ^3, 9, 10^. Most of them are incidentally identified during screening endoscopy or imaging studies because they are usually asymptomatic. However, just like any other GI tumors, they can present some clinical symptoms such as abdominal pain, tenesmus, rectal bleeding or melena ^1^. Sometimes these tumors manifest as colonic obstruction or intussusception ^4, 9, 10^.

Endoscopically, these tumors usually present as a submucosal tumor with smooth mucosa or with mucosal ulceration ^1– 3^. On CT scans, the tumors usually present as exophytic masses with homogeneous enhancement and cystic change, necrosis, or calcification within tumors are uncommon ^2^.

A preoperative diagnosis is challenging because endoscopic mucosal biopsy usually provides limited information to differentiate them from other mesenchymal tumors of GI tract such as GISTs, NETs, leiomyomas, or leiomyosarcomas ^3^. In Inagawa’s study, only 15% of the colon schwannomas were diagnosed on preoperative endoscopic biopsy ^11^; in Bohlok’s study, 24% of the colorectal schwannomas were diagnosed preoperatively ^3^.

Diagnosis is confirmed pathologically with immunohistochemical analysis. Histopathological features of schwannomas are mainly elongated bipolar spindle cells with variable cellularity and sometimes peripheral cuff-like lymphocyte infiltration is exhibited around the tumor, which helps to differentiate schwannomas from other spindle-cell tumors like fibromas or leiomyomas ^5, 7, 11^. Schwannomas can be distinguished from other smooth-muscle tumors by strong s-100 positivity in immunohistochemical analysis ^11– 13^. Additionally, CD34 or c-kit protein is useful to distinguish the schwannomas from GISTs ^11– 13^. Schwannomas are S-100 positive, but CD-34 and c-kit negative; most GISTs are s-100 negative, but CD-34 and c-kit positive.

Prognosis is generally promising because most of the GI schwannomas are benign and malignant potential is low ^1– 3^. However, even though many researchers reported the benign features of GI schwannoma, some of these tumors present local recurrence or distant metastasis. In Bohlok’s study, 3 (3.1%) out of 93 cases of colorectal schwannomas were malignant ^3^. High mitosis rate, high Ki-67 index, and large tumor size are considered to be associated with malignancy ^3^.

Complete surgical resection obtaining free resection margins is the best therapeutic option ^1, 3^, because tumor recurrence is generally owing to incomplete surgical resection with inadequate margins ^2^. In some limited cases, patients can be treated by endoscopic resection or transanal resection without undergoing radical surgery ^3^.

Adjuvant therapies are not commonly recommended if surgical resection achieving free margins is completed ^5, 14^. Currently, limitation of our knowledge is that there is no consensus for subsequent treatment after surgical resection in case of malignant transformation ^5^.

Sigmoid colon schwannomas are very rare colonic neoplasms. To our knowledge, only 28 cases of sigmoid colon schwannomas have been published. Because of its rarity and challenge to diagnosis, review of the clinical features of the disease with a presenting case would be of help for physicians and surgeons. We believe that our study presents the clinical manifestations including endoscopic, imaging, histopathologic and immunohistologic findings of this rare disease with a thorough literature review and it provides guidance in diagnosis and treatment of the disease. Limitation of this study is that the treatment strategy for metastatic diseases was not suggested because only very limited cases were reported and no consensus exists for now.

## Conclusion

Sigmoid colon schwannomas are usually found incidentally during screening colonoscopy and present as submucosal tumors. Preoperative diagnosis is challenging because clinical manifestations, as well as colonoscopic and CT findings, are nonspecific. No specific tumor marker exists either. Histopathologically, the tumors consist of spindle cells. However, colonoscopic biopsies have limitations in terms of a definite diagnosis and differential diagnosis includes schwannoma, GIST, NET, leiomyoma, leiomyosarcoma, etc. Conclusive diagnosis can be made by confirming s-100 proteins in immunohistochemical analysis and mostly confirmed post-surgically. Complete surgical resections with adequate free margins are required because although the majority of the diseases are benign, some are reported to be malignant. There is no consensus for adjuvant chemotherapy.

## Data availability

### Underlying data

All data underlying the results are available as part of the article and no additional source data are required.

## Consent

Written informed consent for publication of their clinical details and clinical images was obtained from the patient.
