# Supplementary material for: Case Report: Schwannoma of the sigmoid colon: a case report of a rare colonic neoplasm and review of literature
Source: F1000Res. 2019 May 13;8:652. [Version 1] doi: 10.12688/f1000research.19110.1 (PMC6777014; doi:10.12688/f1000research.19110.1)
Supplement: Peer review history

# Peer review: Reviewer response for version 1

**DOI:** 10.5256/f1000research.20942.r54272

**Published:** 2019-10-02

**Authors:** Umut Riza Gunduz

**Affiliations:** 1 Department of General Surgery, University of Health Science, Antalya Training and Research Hospital, Antalya, Turkey

The authors presented a case report and a review of related literature about sigmoid colon schwannomas. This is a rare disease and an interesting subject.

 Although it is a beautifully written review in general, some corrections are required:

- The authors claimed segmenter resection is enough for sigmoid colon schwannoma but in their case report they prefer to anterior resection. it is necessary to explain this in the discussion section.
- The authors shared a photo of the specimen they resected in figure 3. This image resembles segmental resection rather than anterior resection.

I confirm that I have read this submission and believe that I have an appropriate level of expertise to confirm that it is of an acceptable scientific standard.

# Peer review: Reviewer response for version 1

**DOI:** 10.5256/f1000research.20942.r48558

**Published:** 2019-05-31

**Authors:** Huaibin Mabel Ko

**Affiliations:** 1 Division of Gastroenterology, Department of Medicine, Icahn School of Medicine at Mount Sinai, New York, NY, USA

Overall this is a good case report and review of a rare tumor. There are a few small grammatical errors, but the paper is otherwise well organized and easy to follow.

**Minor suggestions:**

1. When referring to the size of the tumor, especially when giving dimensions that seem fairly exact ("4.5 x 4.0 cm") it is unnecessary to say "about", you can just state the size.
2. Replace "Most of the" with "Most", as in "Most GI tract schwannomas" or "Most colon schwannomas"
3. Figure 6: "proper muscle layer" should be "muscularis propria layer"

I confirm that I have read this submission and believe that I have an appropriate level of expertise to confirm that it is of an acceptable scientific standard.
